# Synthesis of Novel pH-Tunable Thermoresponsive Hydroxyl-Terminated Hyperbranched Polyether

**DOI:** 10.3390/polym11050895

**Published:** 2019-05-16

**Authors:** Xiuzhong Zhu, Xiao Duan, Ting Bai, Xuan Zhang, Tong Wang, Tao Cao, Xiaodong Fan

**Affiliations:** 1 The Key Laboratory of Space Applied Physics and Chemistry, Ministry of Education and Shaanxi Key Laboratory of Macromolecular Science and Technology, School of Science, Northwestern Polytechnical University, Xi’an 710072, China; zhuxiuzhong321@163.com (X.Z.); baiting_zoe@mail.nwpu.edu.cn (T.B.); dgjq170289@163.com (X.Z.); 17795837806@163.com (T.W.); caotao@mail.nwpu.edu.cn (T.C.); 2 Department of Pharmceutical analysis of Changzhi Medical College, Changzhi 046000, China; duanxiao0211@outlook.com

**Keywords:** hydroxyl-terminated hyperbranched polyether, cationic polymerization, thiol-ene “click” reaction, widely tunable LCST

## Abstract

In this study, a new pH-tunable thermoresponsive hydroxyl-terminated hyperbranched polyether (HTHP 2) was successfully prepared via a one-pot cationic polymerization technique and postmodification. In the first step, hydroxyl-terminated hyperbranched polyether containing double bonds (HTHP 1) were synthesized. Then, through thiol-ene “click” reaction, pH-responsive carboxyl groups were introduced to the target polymer of HTHP 2. The products were characterized via Fourier-transform infrared spectra (FTIR), nuclear magnetic resonance (NMR), and size-exclusion chromatography-multiangle laser light scattering (SEC-MALLS). Moreover, dynamic light scattering (DLS) and UV–Vis spectroscopy was employed to study the pH- and thermoresponsiveness in detail. Results showed that HTHP 2 possessed typical pH-controllable thermoresponsive behavior. By regulating the solution pH value range 3.0–5.4, LCST of HTHP 2 could be changed from 12.8 to 68.0 °C. Meanwhile, the cell viabilities of A549 cells were more than 80% for in vitro cytotoxicity tests of HTHP 2, suggested that HTHP 2 polymers are of good biocompatibility for up to 24 h.

## 1. Introduction

Thermosensitive polymers with lower critical solution temperature (LCST), such as poly(*N*-isopropylacrylamide) (PNIPAAM), poly(*N*-vinylcaprolactam), poly(2-oxazoline)s, poly(2-oxazine)s, oligo(ethylene glycol)-containing polymers, and hyperbranched polymers, have been widely studied in academia and industry [1,2,3,4,5,6,7,8,9,10,11]. Because of their wide application in materials science and biology, thermosensitive polymers have attracted more and more attention [12,13,14,15]. LCST-type thermosensitive polymers are soluble at low temperature circumstance due to hydrogen bonding with surrounding water molecules, resulting in limited intramolecular/intermolecular interactions between polymer chains. Upon heating above LCST, because of the disruption of the interactions between the polymer and solvating water molecules, the hydrogen bonds are disrupted, and the polymer chains present a phase transition with a reduced solubility [16]. Many studies have shown that the LCST is dependent on many external parameters, including monomer composition, molecular weight, concentration, and the ionic species present. It is significant to develop thermoresponsive polymers with tunable LCSTs in a wide range by external stimuli [17,18,19,20]. However, typically, to have different LCST values, a series of polymers must be synthesized. pH-responsive polymers have functional groups capable of donating or accepting protons upon pH change, which accompanies reversible conformational changes between the extension and collapse state. The small variations with pH value could result in remarkable changes in hydrophilic/hydrophobic balance of those polymers [21,22,23,24,25]. Therefore, the synthesis of temperature and pH dual-responsive polymers has been a hot topic for scientific research that different LCST values could be obtained by adjusting the pH values.

Nowadays, temperature and pH dual-responsive polymers are mainly block copolymers, that is, one segment of a block copolymer with pH-stimulating behavior is connected with another segment of a block copolymer with temperature-stimulating behavior [26,27,28,29]. Meanwhile, it usually takes a long time to prepare and purify block copolymers to obtain well-defined pure block copolymers. In addition, some block copolymers self-assemble in solution due to the incompatibility between blocks. Therefore, the preparation of homopolymers with both pH and temperature responsiveness, such as poly(2-(dimethylamino) ethyl methacrylate) (PDMAEMA) homopolymers, has become a long-term research goal [30,31]. A feasible solution is to synthesize small molecules with both thermal and temperature responsive groups, and then prepare homopolymers with clear thermal- and pH-responsive groups in each repeating unit by monomer polymerization. For example, Van Hest, and Li et al. reported the successful synthesis and property of temperature and pH-responsive homopolymers [32,33]. However, the preparation of small molecules in this method was complex, which limited the variety of temperature and pH dual-reactive polymers. As an alternative strategy, postpolymerization modification is an effective method for the preparation of new polymer species. For example, a series of pH-tunable thermoresponsive PEO-based functional polymers were prepared by thiol-ene “click” reaction [16]. In addition, Zhang and coworkers reported the thiol-ene modification of polybutadiene to obtain pH-responsive polyethylene derivatives using visible light, showing that all double bonds were converted in a short time [34]. Regretful, no matter small molecule modification or postpolymerization modification, the synthesis of temperature and pH dual-responsive polymers mainly confined to the linear or brush polymer. The preparation of hyperbranched polymers both temperature and pH dual-responsive, especially hydroxyl-terminated hyperbranched polyether are rarely reported.

Hydroxy-terminated hyperbranched polyether (HTHP) is a novel polymeric material, containing a large number of terminal hydroxyl groups [35,36,37]. Due to its excellent biocompatibility, HTHP is widely employed in biological materials, adhesives, etc. [38,39,40,41,42] Especially, in case of drug delivery systems, HTHP with unique highly branched molecular structures, is able to improve the disadvantages of linear amphiphilic polymeric with low encapsulation efficacy, low drug loading efficiency and low efficacy [43,44,45]. Therefore, hyperbranched polymer of HTHP with temperature and pH dual-responsiveness have the significant advantage compared to liner polymers.

**Figure 1:**
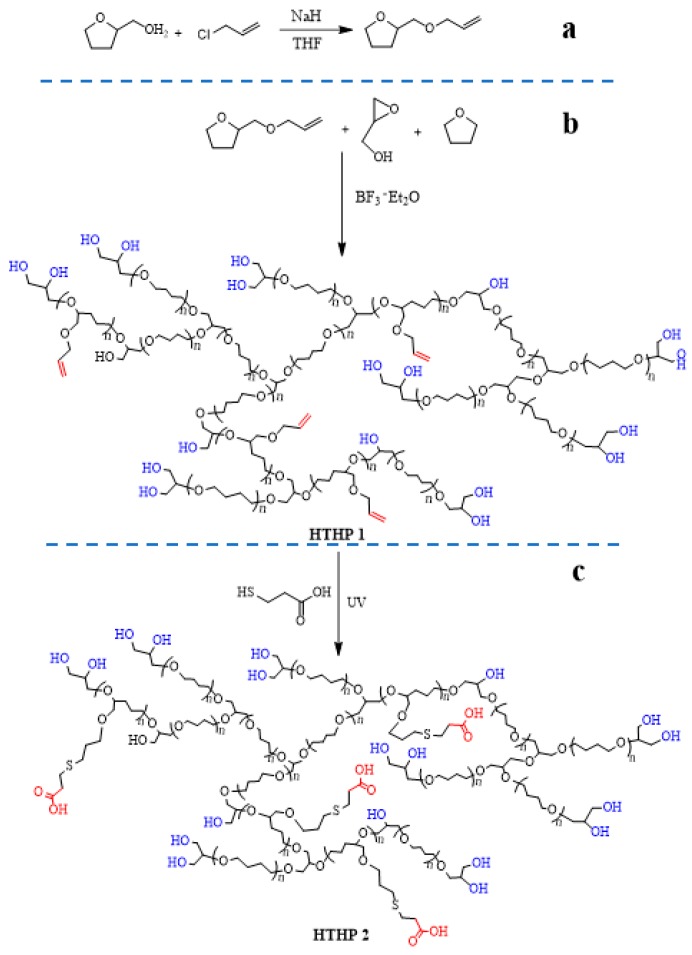
The synthesis route of monomer AMTHF (**a**), HTHP 1 (**b**), and HTHP 2 (**c**).

Here, we synthesized a novel pH-Tunable thermoresponsive HTHP with widely range of LCST values in different pH environment. Based on the previous work [46], a functional monomer, 2-(allyloxy methyl)tetrahydrofuran (AMTHF, as shown in Figure 1a), was synthesized and copolymerized with tetrahydrofuran (THF) and glycidyl to successfully obtain the backbone-thermoresponsive HTHP 1 by one-pot cationic polymerization(as shown in Figure 1b). This novel HTHP contained a large number of double bonds, which could be reacted with thiol groups by thiol-ene “click” reaction. Hence, a new polymer named HTHP 2 with pH and thermo dual-responsiveness was successfully synthesized through thiol-ene reaction (as shown in Figure 1c). The structures of HTHP 1 and HTHP 2 were characterized by nuclear magnetic resonance (NMR) spectroscopy and size-exclusion chromatography (SEC). Then, we focused on the study about the LCST values of HTHP 2 by changing pH value and its biocompatibility.

## 2. Experimental Sections

### 2.1. Materials

Tetrahydrofurfuryl alcohol (97%, Alfa Aesar, Shanghai, China), 3-allyl chloride (98%, Alfa Aesar, Shanghai, China), tetrahydrofuran (99.9%, Sinopharm Chemical Reagent Co., Ltd, Tianjin, China), and glycidol (96%, Alfa Aesar, Shanghai, China) were removed water using molecular sieve. Boron trifluoride etherate (48%, Acros, BE), 3-Mercaptopropionic acid (98%, Aladdin, Shanghai, China), 2,2-dimethoxy-2-phenylacetophenone (99%, Acros, BE), and sodium hydride (50%, Sinopharm Chemical Reagent Co., Ltd, Tianjin, China) were used as received. Diethyl ether (99%, Sinopharm Chemical Reagent Co., Ltd, Tianjin, China) other organic solvents were all purchased from Sinopharm Chemical Reagent Co., Ltd (Tianjin, China) and used without any purification.

### 2.2. Characterization

Fourier-transform infrared (FTIR) spectra were obtained on a Nicolet iS10 FT-IR instrument (Nicolet Instrument Corporation, Madison, WI, USA) in the region from 4000 to 500 cm^−1^. The ^1^H NMR and ^13^C NMR spectra were carried out on a Bruker 400 MHz NMR spectrometer (Bruker Corporation, Karlsruhe, Germany) using deuterated chloroform (CDCl_3_) or deuterated dimethylsulfoxide (DMSO-*d*_6_) as solvents, and tetramethylsilane (TMS) as the internal standard. The molecular weights and polydispersity indexes of the polymers were determined on a Wyatt DAWN EOS SEC-MALLS (Santa Barbara, CA, USA), THF was used as the eluent with a flow rate of 0.5 mL/min at 25 °C.

The optical transmittance of polymer solutions at varying pH or temperature values were measured by UV–Vis spectroscopy (Shimadzu UV-2550, Kyoto, Japan) at a wavelength of 500 nm. The zeta potentials of the aggregates were determined by Dynamic light scattering (DLS, Malvern Instruments, Malvern, UK) at different pH or temperature values. The scattered light of a He–Ne laser at 633 nm was measured at an angle of 173°. All the samples were measured directly without any filtration.

The in vitro cytotoxicity of the HTHP and the viability of A549 cells were evaluated by using the cell counting kit (CCK-8) assay. The A549 cells in Dulbecco‘s modified Eagle‘s medium (DMEM) supplemented with 10% fetal bovine serum (FBS ) were seeded into 96-well plates at a density of 1 × 104 cells per well and cultured for 24 h at 37 °C under CO2/air (5/95, *v*/*v*). Then, the cells were cultured with a medium containing various concentrations of HTHP from 50 to 500 μg/mL. After the cells were incubated for 24 h, 10 μL of CCK-8 solution was added to each well and the cells were incubated for another 2 h at 37 °C. Cell viability was determined by using a microplate reader of absorbance at 450 nm.

### 2.3. Synthesis of Monomer AMTHF

Tetrahydrofurfuryl alcohol (9.7 mL, 0.1 mol) and THF (100 mL) were added into a dry 500 mL three-necked flask. After 30 minutes of stirring in an ice bath, NaH (4.8 g, 0.2 mol) was added into the solution slowly under N_2_ atmosphere. The solution was allowed to stir in the ice bath for another 4 h. Then, 3-allyl chloride (24.4 mL, 0.3 mol) was added into the solution. The solution was allowed to stir at room temperature for another 12 h. Finally, the precipitate was filtrated, and the solvent was removed under vacuum using a rotary flash evaporator at 38 °C. The product obtained was distilled again under reduced pressure at 130 °C and 0–5 mmHg pressure to obtain pure AMTHF.

### 2.4. Synthesis of HTHP 1

In a typical reaction (HTHP 1a in Table 1), THF (2 mL), AMTHF (1 mL), and glycidol (0.6 mL) were added into a dry 50 mL single-neck flask. After a few minutes of stirring in an ice bath, boron trifluoride diethyl etherate (BF_3_·OEt_2_, 0.1 mL) was added into the solution slowly. The solution was allowed to stir in the ice bath for another 2 h. Then 2 mL water was added to quench the reaction. The mixture was dialyzed against distilled water (3500 Da) for 36 h, and the water was replaced every 12 h. The obtained solution was lyophilized to obtain a viscous liquid.

### 2.5. Synthesis of HTHP 2

HTHP 1a (300 mg) was dissolved in THF (5 mL) and added into a dry 25ml quartz tube. Under an argon atmosphere, 3-mercaptopropionic acid (200 mL) and 2,2-dimethoxy-2-phenylacetophenone (DMPA, 2 mg) were added. Then, the solution was allowed to stir under UV light for 5 h. Subsequently, the polymer was precipitated in diethyl ether and residual solvent was removed by rotary evaporation. A viscous liquid product (HTHP 2) can be obtained at this stage.

## 3. Results and Discussion

### 3.1. Synthesis and Characterization of Monomer AMTHF

**Figure 2:**
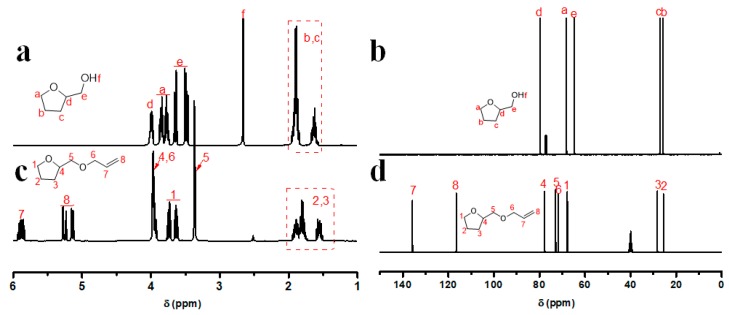
^1^H NMR spectra of tetrahydrofurfuryl alcohol (**a**) and AMTHF (**b**). ^13^C NMR spectra of tetrahydrofurfuryl alcohol (**c**) and AMTHF (**d**).

Route for the synthesis of AMTHF is shown in Figure 1a. It was obtained by condensation of tetrahydrofurfuryl alcohol and chloropropene. Figure 2 shows the ^1^H NMR and ^13^C NMR spectra of tetrahydrofurfuryl alcohol and AMTHF. In Figure 2a, the peak at 2.66 ppm was attributed to the hydroxyl group in tetrahydrofurfuryl alcohol. In Figure 2b, this peak at 2.66 ppm disappeared totally, whereas new peaks for double bond appeared at 5.15–5.96 ppm. This suggested that the hydroxyl groups in tetrahydrofurfuryl alcohol were completely substituted by chloropropene molecules. Moreover, in the ^13^C NMR spectra (Figure 2c,d), the disappearance of peak at 64.7 ppm (methylene adjacent to hydroxyl group) and the appearance of peaks due to double bond at 116.5 and 135.7 ppm in the spectrum of AMTHF further confirmed the successful synthesis of AMTHF.

### 3.2. Synthesis and Characterization of HTHP 1

**Figure 3:**
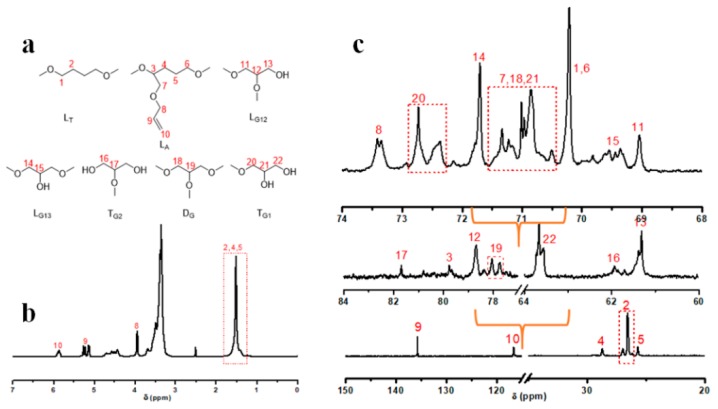
The seven structural units of HTHP 1 (**a**), ^1^H NMR spectrum of HTHP 1 (**b**), and ^13^C NMR spectra of HTHP 1 (**c**).

**Figure 4:**
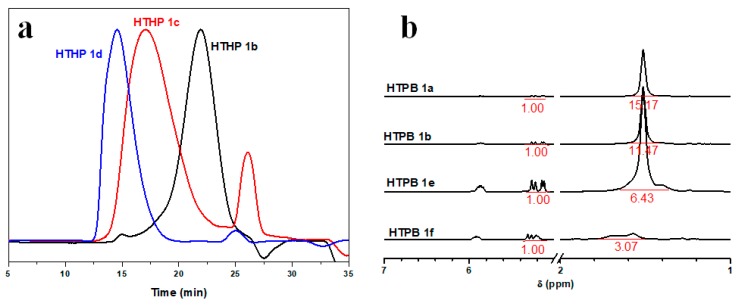
Size-exclusion chromatography (SEC) curves of the HTHP 1 obtained with different rate of charge (**a**) and ^1^H NMR spectra of HTHP 1 with different content of double bond (**b**).

HTHP 1 was synthesized by a one-pot cationic ring-opening copolymerization of AMTHF, THF, and glycidol (Figure 1b). HTHP 1 with different molecular weights were synthesized by adjusting the feed ratios of AMTHF, THF, and glycidol, and the results are presented in Table 1. Moreover, the SEC curve is shown in Figure 4a. The products were purified by dialysis to completely remove the unreacted monomers and small oligomers, so the elution curves were regular and narrow, with no trailing peaks of small molecules. ^1^H NMR and ^13^C NMR techniques were employed to characterize the structures of HTHP 1. As shown in Figure 3b, the peaks at 5.88 and 5.08–5.30 ppm were attributed to the protons of double bond in HTHP 1; the peak at 3.94 ppm was associated to the protons of methylene adjacent to the double bond. In addition, the peaks at 1.29–1.69 ppm were associated to the protons of methylene groups in THF and AMTHF units (–OCH_2_C***H***_2_C***H***_2_CH_2_O–, –OCHC***H***_2_C***H***_2_CH_2_O–). Because there were THF, AMTHF, and glycidol units in the structure of hyperbranched polyether, the glycidol unit was linked in multiple ways, which resulted in the overlapping of corresponding characteristic peaks and made it difficult to integrate the peaks for further analysis. Therefore, quantitative ^13^C NMR analysis was used to further characterize the molecular structures of hyperbranched polyether. By referring to the studies of Yan and Frey, and combining it with our previous research work, it was found that there were seven structural units in the HTHP 1- THF linear unit (L_T_), AMTHF linear unit (L_A_), glycidol branched unit (D_G_), two glycidol linear units (L_G12_ and L_G13_), and two glycidol terminal units (T_G1_ and T_G2_). The structures and corresponding chemical shifts of all units are shown in Figure 3a and Figure 3c, respectively.

The polymerization process for the synthesis of HTHP 1 with different feed ratios of AMTHF, THF, and glycidol was studied. Keeping the feed ratios of THF and glycidol constant, the molecular weight of HTHP 1 increased with increase in feed ratio of AMTHF. The result was shown in Table 1 (sample a, b). In addition, as shown in Figure 3b, the integral ratio of the peaks of HTHP 1a and HTHP 1b (Table 1) at δ = 5.08–5.30 and 1.29–1.69 ppm is 1.00:15.17 and 1.00:10.78, respectively. The results suggested that the content of double bonds in the HTHP 1 increased gradually with increase in AMTHF amount. Keeping the feed ratios of THF and AMTHF constant, the molecular weight of HTHP 1 increased with increase in feed ratio of glycidol. This was shown by the successive decrease in the elution time of the samples as measured by the differential detector (Figure 4a and sample b–d in Table 1). The main reason for this result maybe was that glycidol acted as the promoter for initiation of the ring opening of THF and AMTHF during copolymerization, so that a greater number of THF and AMTHF molecules could participate in the polymerization process with increase in feed ratio of glycidol. Moreover, in the cationic ring-opening polymerization system, a large number of hydroxyl groups were present. The active centers not only transferred to the hydroxyl groups, but also captured THF and AMTHF molecules for ring-opening polymerization. These two processes competed with each other. However, due to the high strain of glycidol ring, its cationic ring opening process was hardly affected by the hydroxyl groups [46,47,48]. Hence, increase in the feed ratio of glycidol could improve the monomer conversion, which resulted in HTHP 1 with higher molecular weights. Keeping the glycidyl content unchanged, and the total amount of THF and AMTHF the same, just adjusting the volume ratio of THF and AMTHF, the content of double bonds in the HTHP 1 increased gradually with increasing AMTHF amount. This result could be confirmed by the ^1^H NMR spectra. As shown in Figure 3b, we have already pointed out that the peaks at 5.08–5.30 ppm was attributed to the double bond in HTHP 1, and the peaks at 1.52 ppm was attributed to the methylene in L_T_ and L_A_ units (Figure 3a,b). Therefore, the content of double bonds in the HTHP 1 could be obtained through calculating the integral ratio of the peaks at δ = 5.08–5.30 and 1.52 ppm (Figure 4b). As shown in Figure 4b, the integral ratio of the peaks at δ = 5.08–5.30 and 1.52 ppm increased following the increase of AMTHF (sample b, e, and f in Table 1). Besides, the molecular weight of HTHP 1 (sample b, e and f in Table 1) decreased with increase in amount of AMTHF, due to higher ring strain of AMTHF as compared to that of THF.

### 3.3. Synthesis and Characterization of HTHP 2

**Figure 5:**
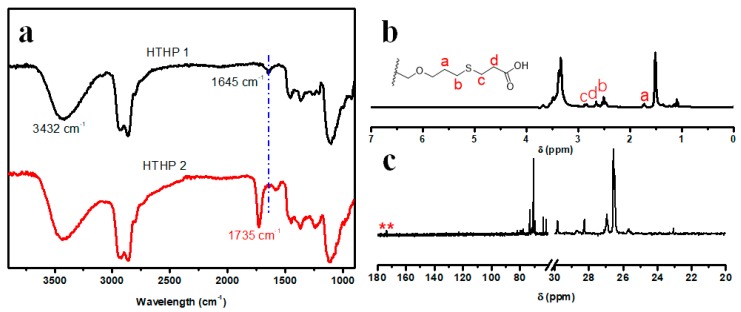
FTIR spectra of HTHP 1 and HTHP 2 (**a**), ^1^H NMR spectrum of HTHP 2 (**b**), and ^13^C NMR spectrum of HTHP 2 (**c**).

UV-induced thiol-ene “click” reaction was used to synthesize HTHP 2 (Figure 1c). HTHP 1a (Sample a in Table 1) and HTHP 1b (Sample b in Table 1) were reacted with 3-mercaptopropionic acid to obtain HTHP 2a and HTHP 2b, respectively. Using HTHP 2a as an example, Figure 5a shows the FTIR spectra of HTHP 1a and HTHP 2a, the disappearance of the peak for double bond at 1645 cm^−1^ and appearance of peak due to carboxyl group at 1735 cm^−1^ confirmed the complete consumption of the double bonds and successful introduction of carboxyl groups. Moreover, complete disappearance of peaks between 5.15 and 5.96 ppm in the ^1^H NMR spectrum corresponding to the double bond and the appearance of peak due to methylene protons (2.56–2.73 ppm) adjacent to 3-mercaptopropionic acid group further suggested the successful synthesis of HTHP 2 (Figure 5b). Furthermore, the ^13^C NMR spectrum of HTHP 2a was shown in Figure 5c. Disappearance of peaks due to double bonds at 115 and 138 ppm and the appearance of peaks for carboxyl groups at 176 ppm also suggested that all the double bonds in HTHP 1a were involved in the thiol-ene “click” reaction to successfully synthesize HTHP 2a. Therefore, based on the above spectral analyses, we infer that carboxyl groups have been efficiently grafted to the backbone of HTHP 2 via the thiol-ene reaction.

### 3.4. pH-Responsive Behavior of HTHP 2

**Figure 6:**
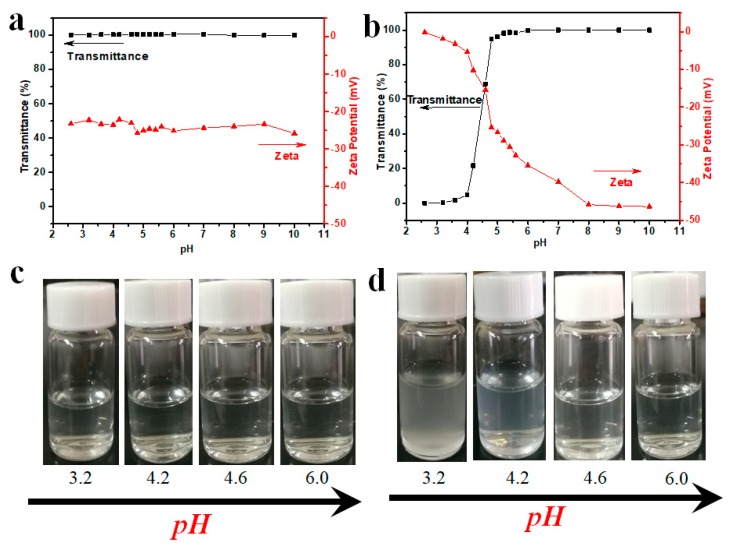
Transmittance and zeta potential of HTHP 1a (**a**) and HTHP 2a (**b**) with the concentration of 2.0 mg/mL at different pH values; turbidity change of HTHP 1 (**c**) and HTHP 2 (**d**) with the concentration of 2.0 mg/mL at different pH values.

Traditional HTHP do not contain any ionizable groups and so they are not pH responsive. On incorporation of appropriate number of ionizable groups, HTHP exhibit pH responsiveness accordingly. As shown in Figure 6a,c, since HTHP 1 contained no ionizable group, the transmittance and zeta potential of HTHP 1 solution showed little variations with change in pH value. In case of HTHP 2 (Figure 6b,d), due to the incorporation of carboxyl group, it could ionize to generate H^+^ at suitable pH value, and so it showed good pH responsiveness. When the pH was decreased from 10 to 4.8, the transmittance of HTHP 2 solution almost remained unchanged, with only a decrease from 100% to 94.8%. However, when the pH was decreased slightly from 4.8 to 4, the transmittance of HTHP 2 solution showed drastic decrease from 94.8% to 4.7%. Meanwhile, its zeta potential dropped from −46.3 to −15.4 mV, and finally to −0.2 mV. This was due to the fact that the carboxyl group was completely disassociated accompanied with increase in hydrophilicity of HTHP 2 at high pH value. When the pH value decreased to 4, carboxyl groups showed increase in protonation due to increase in hydrophobicity of HTHP 2. As a result, the transmittance dropped sharply. Meanwhile, due to protonation of carboxyl group, there was a reduction in zeta potential all through. 

### 3.5. Thermoresponsive Behavior of HTHP 2

**Figure 7:**
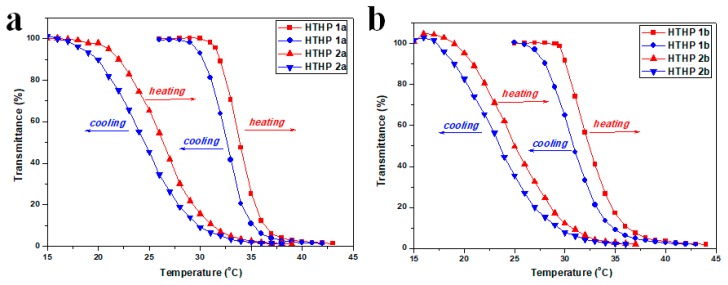
Temperature dependence of the transmittance of HTHP 1a and HTHP 2a (**a**) and HTHP 1b and HTHP 2b (**b**), with the concentration of 2.0 mg/mL during one heating and cooling cycle.

Thermoresponsive phase transition behavior of the HTHP was tested by UV–Vis approach as shown in Figure 7. Transmittance changes of HTHP solution (λ = 500 nm) following temperature was measured to determine the LCST, which was defined as the temperature at which a 10% decrease in transmittance. Figure 7 showed the transmittance changes of HTPB 1a, 1b, 2a, and 2b solution during heating and cooling processes, respectively. They all obviously displayed a reversible and abrupt change in transmittance during the course of heating and cooling. However, the hysteresis was observed clearly in a heating-and-cooling cycle, attributed to the formation of additional hydrogen bonds of each primary ether bond or carboxyl groups that inhibits hydration in the cooling process. In the meantime, it was easy to observe the sharp transition between a transparent and cloudy solution with increasing temperature (Figure 9d, using HTHP 1a for example). From Figure 7, we could also find that the LCSTs of those HTHP were different. For HTHP 1a (7560 g/mol) and HTHP 1b (8420 g/mol), their LCSTs were 31.9 and 30.1 °C, respectively. Compared with HTHP 1a, HTHP 1b had lower LCST, that the main reason may be attributed to increasing the lyophobic chain with AMTHF units in the backbone of HTHP 1b. Besides, the LCSTs of HTHP 2a and HTHP 2b were 22.0 and 21.9 °C, respectively, which was also lower than HTHP 1a and HTHP 1b, respectively. Those results suggested that hydrophobicity increased after 3-mercaptopropionic acid was introduced into HTHP 1. In previously synthesized hyperbranched polyether from THF and glycidol [46], it was evident that compared to other temperature sensitive polymers, hyperbranched polyether showed a slow phase transition phenomenon. This was due to the random distribution of hydrophobic units of hyperbranched polyether in hyperbranched structures. Hence, from Figure 7, transmittance of HTHP 2 decreased more slowly compared to HTHP 1, depicting a slower phase transition phenomenon. The reason may be that hydrophilicity due to carboxyl group increased with increase in temperature that altered the hydrophilic–hydrophobic equilibrium [49].

**Figure 8:**
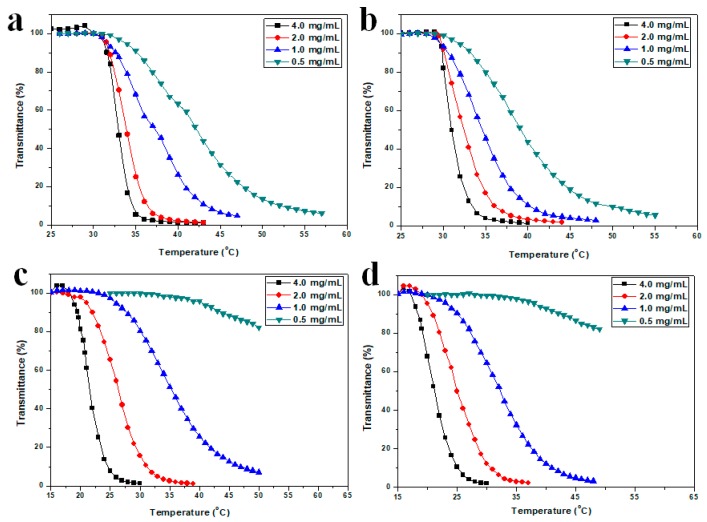
Temperature dependence of the transmittance of HTHP 1a (**a**), HTHP 1b (**b**), HTHP 2a (**c**) and HTHP 2b (**d**) with different concentrations.

In order to test polymer concentration (c_p_) effect on the LCST of the HTHP, the concentration dependence on LCST of HTHP 1a (Figure 8a), HTHP 1b (Figure 8b), HTHP 2a (Figure 8c), and HTHP 2b (Figure 8d) were examined, respectively. As c_p_ dropped from 4.0 to 0.5 mg/mL, LCST (HTHP 1a) gradually shifted from 31.6 to 35.4 °C, LCST (HTHP 1b) increased from 29.9 to 33.2 °C, LCST (HTHP 2a) increased from 19.5 to 43.9 °C, and LCST (HTHP 2b) increased from 18.4 to 42.1 °C. These observations showed that the LCST increased upon lowering the concentration of the polymer, which was consistent with previous reports [50]. In addition, the temperature differences of LCST of HTHP 1a (ΔT_L_ = 3.8 °C) from 4.0 to 0.5 mg/mL were similar with HTHP 1b (ΔT_L_ = 3.3 °C for HTHP 1b), and meanwhile, HTHP 2a (ΔT_L_ = 24.4 °C) and HTHP 2b (ΔT_L_ = 23.7 °C) also had the same results. Those results should be attributed to the same chemical structure of HTHP 1a and HTHP 1b, HTHP 2a, and HTHP 2b, respectively. But, the amplitude of LCST increases for HTHP 2a and HTHP 2b were bigger than HTHP 1a and HTHP 1b, that due to having different ionization degrees of carboxyl groups. As the concentration of HTPB 2a and 2b decreased, the degree of carboxy group ionization increased, which resulted in hydrophilic enhancement of HTPB 2a and 2b. 

**Figure 9:**
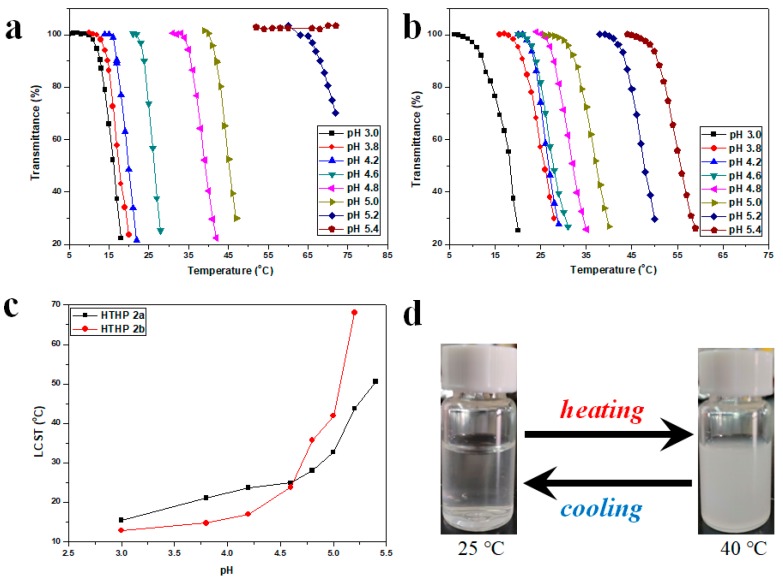
Temperature dependence of the transmittance of HTHP 2a (**a**) and HTHP 2b (**b**) with the concentration of 1.0 mg/mL at different pH values in citric acid–disodium hydrogen phosphate buffer solution, influence of pH on the LCST of the HTHP 2a and HTHP 2b (**c**), turbidity change of HTHP 2a with the concentration of 2.0 mg/mL during heating and cooling cycle. (**d**).

According to the above research, HTHP 2a and HTHP 2b have carboxyl groups and their corresponding hydrophilicities can be affected by the pH values of the aqueous medium. Hence, the thermoresponsive properties should be influenced by the pH values of the aqueous medium. In order to expound this phenomenal, the thermally induced phase transition behaviors of HTHP 2a and HTHP 2b were investigated in citric acid–disodium hydrogen phosphate buffer solution at different pH values. Figure 9 represents the transmittance against temperature curves for HTHP 2a and HTHP 2b at 1.0 mg/mL in citric acid–disodium hydrogen phosphate buffer solution at different pH values corresponding to the heating processes. One can see that LCSTs of HTHP 2a were 12.8, 14.7, 16.9, 23.8, 35.6, 41.9, and 68.0 °C at pH of 3.0, 3.8, 4.2, 4.6, 4.8, 5.0, and 5.2, respectively (Figure 9a). When the pH value was increased to 5.4, the transmittance always kept around 100% over the experimental temperature range even at 70 °C. Meanwhile, the LCSTs of HTHP 2b were 15.2, 21.0, 23.7, 24.9, 27.9, 32.5, 43.4, and 50.7 °C at pH of 3.0, 3.8, 4.2, 4.6, 4.8, 5.0, 5.2, and 5.4, respectively (Figure 9b). As the pH was enhanced (3.0–5.4), the LCSTs of HTHP 2a and HTHP 2b gradually increased and even disappeared, those result impacted that the hydrophily of HTHP 2a and HTHP 2b was increased with the increase in pH value. Nonetheless, it should be noted that the LCSTs of HTHP 2a and HTHP 2b were different under same pH value (Figure 9c). At low pH value below 4.6, the LCSTs of HTHP 2a were higher than HTHP 2b; upon increasing above 4.6, the result then was a swift reversal that the LCSTs of HTHP 2a were lower than HTHP 2b. The reason should be attributed to different ionization degrees of carboxyl groups. Because the pKa of 3-mercaptopropionic acid is 4.32, and meanwhile compared with HTHP 2a, HTHP 2b contained more carboxyl groups. When the pH values were lower than 4.32 (pH 3.0, 3.8, and 4.2), the hydrophobic carboxyl groups were the main existing style in polymer structure, which caused the hydrophobic property of HTHP 2b better than HTHP 2a; when the pH values were higher than 4.32 (pH 4.6), part of carboxyl groups were ionized which caused the LCSTs of HTHP 2a and HTHP 2b were similar. As the pH values continued to rise (pH 4.8, 5.0, 5.2, 5.4), the carboxyl groups was further ionized, result that the hydrophilicity of HTHP 2b was better than HTHP 2a.

### 3.6. In Vitro Cytotoxicity of HTHP 1 and HTHP 2

**Figure 10:**
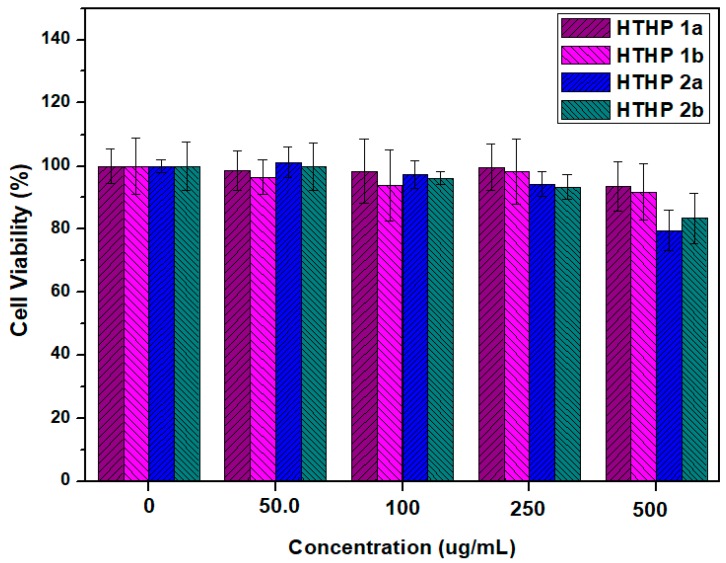
The cell viability of A549 cells after incubation with HTHP 1a, HTHP 1b, HTHP 2a, and HTHP 2b for 24 h.

For potential biomaterials applications, it is necessary to evaluate the toxicity of the novel polymers of HTHP 1 and HTHP 2. The results showed that the critical micelle concentration (CMC) of HTHP 1a, HTPB 1b, HTHP 2a, and HTHP 2b in water which is determined by a fluorescence technique (the excitation wavelength was 335 nm), is 7.90, 9.64, 11.43, and 13.83 ug/mL, respectively. We, thus, evaluated the in vitro cytotoxicity of micelles of HTHP 1a, HTHP 1b, HTHP 2a, and HTHP 2b with different administered concentration ranging from 50 to 500 μg/mL in A549 cells by performing the CCK-8 assay. As shown in Figure 10, the cell viabilities after incubation for 24 h with HTHP 1a and HTHP 1b are both more than 90%, even at a high concentration of 500 μg/mL, suggesting much lower cytotoxicity of HTHP 1a and HTHP 1b. Although HTHP 2a and HTHP 2b due to the carboxyl groups possess a higher cytotoxicity than HTHP 1a and HTHP 1b, when the concentration is more than 250 μg/mL, the cell viabilities of A549 cells were more than 80% with the concentration 500 μg/mL, indicating that HTHP 2a and HTHP 2b also showed a low cytotoxicity. The in vitro evaluations confirmed that HTHP 1 and HTHP 2 are all highly biocompatible for up to 24 h and may be regarded as biomedical materials.

## 4. Conclusions

The synthesis of HTHP with both pH responsiveness and backbone-thermoresponsiveness was reported. Firstly, backbone-thermoresponsive HTHP 1 was synthesized by one-pot cationic polymerization. Then, carboxyl groups were introduced into the side chains by UV-induced thiol-ene “click” reaction. The pH- and thermoresponsiveness of the synthesized polymers were studied in detail. With decrease in pH value, transmittance and the absolute zeta potential values of HTHP 2 decreased significantly, showing an obvious pH-responsiveness. For thermoresponsiveness, incorporation of carboxyl groups into HTHP 2 resulted in a slower phase transition behavior compared with HTHP 1. Meanwhile, by changing the pH values of HTHP 2 solution from 3.0 to 5.4, the LCST increased from 12.8 to 68.0 °C. Moreover, the in vitro evaluations confirmed that HTHP 1 and HTHP 2 were less cytotoxic for up to 24 h and may be regarded as biomedical materials.

## Figures and Tables

**Table 1:** Molecular structural parameters of HTHP 1 with different rate of charge.

Sample	Feed Ratio THF:AMPTHF:glycidol (mL:mL:mL)	*M*_n_^a^ (g/mol)	*M*_w_^a^ (g/mol)	*M*_w_/*M*_n_^a^
a	2.0:0.5:0.6	7600	10,700	1.41
b	2.0:1.0:0.6	8400	11,100	1.32
c	2.0:1.0:0.8	10,500	15,100	1.44
d	2.0:1.0:1.0	12,400	18,800	1.51
e	1.5:1.5:0.6	6000	8800	1.46
f	1.0:2.0:0.6	5400	7700	1.42

^a^ Measured by size-exclusion chromatography-multiangle laser light scattering (SEC-MALLS); THF was used as the eluent with a flow rate of 0.5 mL/min at 25 °C.
